# Impact of Aerobika^®^ oscillating positive expiratory pressure in improving small airway resistance, lung function, symptoms and exercise capacity in chronic obstructive pulmonary disease

**DOI:** 10.3389/fmed.2023.1202380

**Published:** 2023-06-02

**Authors:** Siti Nurhanis Sahardin, Mas Fazlin Mohamad Jailaini, Nik Nuratiqah Nik Abeed, Andrea Yu-Lin Ban, Ng Boon Hau, Azat Azrai Azmel, Shamsul Azhar Shah, Mohamed Faisal Abdul Hamid

**Affiliations:** ^1^Respiratory Unit, Faculty of Medicine, Universiti Kebangsaan Malaysia, Kuala Lumpur, Malaysia; ^2^Department of Community Medicine, Faculty of Medicine, Universiti Kebangsaan Malaysia, Kuala Lumpur, Malaysia

**Keywords:** Aerobika^®^, chronic obstructive pulmonary disease, impulse oscillometry, oscillating positive expiratory pressure, small airway disease

## Abstract

**Background:**

Aerobika^®^ oscillating positive expiratory pressure (OPEP) device promotes airway clearance in many respiratory diseases. However, studies have yet to focus on its effectiveness in improving small airway resistance *via* impulse oscillometry (IOS) measurement in COPD subjects. We aim to evaluate the improvement of small airway resistance (*via* IOS), lung function (spirometry), exercise capacity [*via* 6-min walking test (6MWT)], symptoms [COPD assessment test (CAT)] and severe exacerbation events among COPD subjects using Aerobika^®^ OPEP.

**Methods:**

This was a prospective, single-arm interventional study among COPD subjects with small airway disease. Subjects were instructed to use twice daily Aerobika^®^ OPEP (10 min each session); for 24 weeks; as an additional to standard therapy. IOS, spirometry, 6MWT, CAT score and severe exacerbation events were evaluated at baseline, 12 weeks and 24 weeks.

**Results:**

Fifty-three subjects completed the study. Aerobika^®^ usage showed improvement of IOS parameters; e.g. measurement of airway resistance at 5 Hz (R5), cmH20/L/s, (12-week *p* = 0.008, 24-week *p* < 0.001), R5% predicted (12-week *p* = 0.007, 24-week *p* < 0.001) and small airway resistance (R5–R20), cmH20/L/s, (12-week *p =* 0.021, 24-week *p* < 0.001). There were improvement of lung function; e.g. FEV_1_, L (12-week *p* = 0.018, 24-week *p* = 0.001), FEV_1_% predicted (12-week *p* = 0.025, 24-week *p* = 0.001), FEF_25–75_, L (12-week *p* = 0.023, 24-week *p* = 0.002), and FEF_25–75_% predicted (12-week *p* = 0.024, 24-week *p* < 0.001). CAT score improved at 12 weeks (*p* < 0.001) and 24 weeks (*p* < 0.001). Subjects had improved exercise capacity (6MWT, metres) after 24 weeks (*p* = 0.016). However, there was no significant difference in severe exacerbation events 24 weeks before and after Aerobika^®^ usage.

**Conclusion:**

Aerobika^®^ OPEP demonstrated significant improvement in small airway resistance as early as 12 weeks of usage, with sustained improvement at 24 weeks. Aerobika^®^ OPEP administration had significantly improved lung function, 6MWT, and CAT scores over 24 weeks. There was no difference in severe exacerbation events.

## Introduction

Chronic obstructive pulmonary disease (COPD) is the third leading cause of death globally, accounting for approximately 6 percent (%) or more than 3 million deaths in 2012 (1). From 2007 to 2017, the prevalence was estimated to have risen to 15.6% (2). In Malaysia, the prevalence of moderate to severe COPD in 2010 was 4.7% which equals 448,000 cases (3, 4). COPD is characterized by a combination of small airway disease and the destruction of parenchymal lung tissue, resulting in emphysema that impairs gas transfer (5, 6).

Small airways are the primary sites of airflow obstruction in COPD patients, and small airway disease (SAD) is recognized as a functional hallmark of the disease (6–8). The small airway is denoted as airways of less than 2 mm in internal diameter, which lack cartilage and have a substantial proportion of smooth muscles with fewer goblet cells in the epithelial layers (5, 6). 10% to 25% of total airway resistance in healthy lungs is attributable to the small airways, whereas their contribution to total airway resistance increases significantly in COPD. The prevalence of SAD increases progressively with higher COPD global initiative for chronic obstructive lung disease (GOLD) classifications, and it is closely associated with the high disease impact measured by the COPD assessment test (CAT) questionnaire (7, 8). SAD and emphysema play a greater role in the decline of FEV_1_, with the contribution of small airway disease associated with mild-to-moderate COPD. In later stages of COPD (GOLD 3–4), the contributions of small airway disease and emphysema are relatively equal (9, 10). Before 2023, COPD was classified according to 4 groups; GOLD A, B, C, and D (11). In each of GOLD A, B, C, and D class, the prevalence of SAD are 49%, 88%, 61%, and 96%, respectively (7). The GOLD 2023 report, however, categorizes COPD into three groups: A, B, and E (1). The prevalence of SAD is not yet reported following the most recent GOLD classification.

As the small airways are located in the lung periphery, they are difficult to evaluate, which may impede the diagnosis (particularly in early stages), monitoring, detection of responses to clinical interventions, and prognostic evaluation in COPD (12). In lung function evaluation, impulse oscillometry (IOS), a forced oscillation technique, has been proposed as a better SAD detection method than spirometry (7, 8). IOS can also detect COPD’s early stages (7). Despite normal spirometry, IOS may be able to detect abnormal distal airway function (13–15) and subtle airway function changes earlier than conventional spirometry (13, 16, 17). IOS has also been evaluated to predict reversibility in subjects with bronchiectasis (14). However, certain clinical conditions, such as glottal narrowing and buccal air leaks, can resemble small airway resistance from IOS (18).

IOS measures airway resistance and reactance to assess lung function without special manoeuvres (13, 14). At the subject’s mouth, pressure-flow oscillations are superimposed on tidal breaths to measure respiratory system resistance and reactance at various oscillation frequencies (13). It uses sound waves to detect airway changes rapidly. These pressure signals, when analysed, quantify the degree of obstruction in the central and peripheral airways independently (13). IOS enables the measurement of resistance at 5 Hz represents total respiratory resistance (R5) and resistance at 20 Hz (R20) represents the proximal respiratory resistance or larger airway, with the difference between R5 and R20 (R5–R20) indicating small airway resistance (13). Reactance at 5 Hz (X5) indicates tissue elasticity which predominates at peripheral airway, and area of reactance (AX) which is area under the curve represents the total reactance at all frequencies between 5 Hz and resonant frequency (Fres) (13). In the ECLIPSE trial, they had validated baseline IOS measurements for small airway impedance for all, in which COPD subjects had distinct IOS baseline from non-smoker and smoker control subjects (19). Despite observing an impaired respiratory impedance value in the group as a whole, there were still some COPD patients with normal respiratory resistance and reactance. Therefore, neither IOS nor any assessment of respiratory system impedance can be used as a replacement for spirometry in the diagnosis of COPD and determination of underlying severity of airflow limitation (19).

Early detection of SAD in COPD allows the physician to initially provide patients with a more targeted approach to therapy, such as using small particle size inhalers or mechanical intervention, such as oscillating positive expiratory device (OPEP). OPEP device has been recognized as a supplement to conventional airway clearance techniques (20, 21). It has been used to facilitate the clearance of respiratory secretions in patients with impaired coughing ability, especially those with chronic diseases (22). OPEP will provide a linear pathway through an inhalation valve during inhalation. Upon exhalation, a one-way valve within the device mechanism intermittently opens and closes, resulting in positive pressure that holds airways open and sustains expiratory flow (21, 22). When mucus combines with airway vibrations or oscillations that resonate at a similar frequency to the cilia, its viscoelastic properties are diminished (21–23). This will mobilize sputum, making removing it easier by deep force exhalations or coughing (huffing) techniques (21). There are few OPEP available, e.g., Acapella^®^, Flutter^®^, Lung Flute^®^ and Aerobika^®^ that have been studied in subjects with COPD. These devices have been shown to improve lung function (FEV_1_ and FVC), St George respiratory questionnaire (SGRQ), patient evaluation questionnaire (PEQ)-ease-bringing-up-sputum, CAT score, 6-min walking test (6MWT) and reduce moderate-severe exacerbation event (21, 23–27).

The clinical importance of Aerobika^®^ OPEP (Trudell Medical International, Canada) to improve small airway disease in COPD subjects has not yet been widely studied. In this study, our primary objective was to assess the change of small airway parameters using IOS at 12 and 24 weeks after the introduction of Aerobika^®^ OPEP among COPD subjects. Our secondary objectives were to evaluate changes in lung function (from spirometry), symptoms burden (from CAT score), and exercise capacity (from 6MWT) at 12 and 24 weeks of Aerobika^®^ OPEP intervention, as well as to compare the frequency of severe exacerbation and hospital admission; 24 weeks before and after Aerobika^®^ usage. We hypothesize that Aerobika^®^ OPEP helps to improve small airway resistance, lung function, exercise capacity and symptoms, and reduce the severe exacerbation event in COPD subjects.

## Materials and methods

### Study design

This was a prospective, single-arm interventional design study of outpatient COPD subjects in the Faculty of Medicine, Universiti Kebangsaan Malaysia (UKM), Hospital Canselor Tuanku Muhriz; between March 2022 to February 2023. The study was approved by the Research Ethics Committee, Universiti Kebangsaan Malaysia, FF-2021-175, and registered with the clinical trial number on 14/03/2022 (NCT05420740). Written informed consent was obtained from all subjects before enrolment in this study according to international guidelines.

The primary variable outcome in our study was the difference between R5 and R20 (R5–R20, cmH_2_O/L/s). Based on the previous study, we aimed to detect a moderate effect size of 0.5 improvement at the peripheral airway by administering OPEP to the participants (15). Therefore, it required a sample size of 34 to achieve a power of 80% and a level of significance of 0.05 for detecting an effect size of 0.5 paired differences. The calculation was done by using ‘Statulator: An online statistical calculator. Sample Size Calculator for Comparing Two Paired Means’ (28). An additional 40% of samples were recruited to cover for subject consent withdrawal, dropout, or missing data. Therefore, a total of a minimum of 48 subjects was required for this study.

In our study, subjects were confirmed to have COPD by post bronchodilator FEV_1_/FVC of <0.7 based on COPD GOLD 2023 guideline (1). We defined small airway resistance when they had an IOS parameter of R5–R20 > 1.94 cmH_2_O/L/s (0.19 kPa/L/s) as all our subjects were confirmed to have COPD. This data was taken from COPD subjects in the ECLIPSE trial (19). We converted the unit to cmH_2_O/L/s which is in line with the unit used in our IOS machine. Severe exacerbation in our study subjects defined as worsening dyspnea ± productive cough that required hospitalization.

Other than confirming to have small airway disease by IOS, we included the following subjects: age 40 years and above; moderate to very severe COPD (GOLD 2 to 4); sputum-producer COPD subjects (coughed and brought up sputum more than two days a week or almost every day in the month before the study) (23), able to perform IOS, spirometry and 6MWT.

Subjects were excluded if they had a diagnosis of other chronic lung diseases (such as asthma, asthma-COPD overlap, interstitial lung disease and bronchiectasis); subjects with relative contraindication for spirometry (such as unstable cardiovascular diseases, post-major intrathoracic or intraabdominal surgery, increase pressure in the sinus, middle ear, intracranial or intraocular, or those with infection control issue) (29); subjects with limitation to perform 6MWT (such as a recent acute coronary event, resting tachycardia and hypertension) (30). Other than that, subjects who were not recommended for OPEP (such as neuromuscular weakness, recent facial, oral or skull surgery or trauma, recent oesophageal surgery, active haemoptysis, acute sinusitis, untreated pneumothorax, known or suspected tympanic membrane rupture or other middle ear pathology, overt right-sided heart failure) (21) were also excluded from the study. Any subject that had a change of inhaler during the study period would also be dropped out from the study.

### Methods

Measurement of small airway parameters was done by using *IOS (Carefusion Germany 23X).* The procedure was performed by a staff with adequate training. The subjects were required to breathe in a steady and relaxed manner. Their posture was important, requiring them to be seated in an upright position with correct head position, cheek support, mouthpiece seal, and tongue placement. The nose clip and proper mouth seal were necessary to prevent air leakage during the procedure. Each measurement lasted 30 s, and at least three measurements were taken. The coefficient of variation between replicates was taken as 10% or less to fulfil the acceptability criteria (31). Coherence value is another important parameter used to determine the validity and quality of the test results, it should be ≥0.6 to 0.8 at 5 Hz (32, 33). IOS was conducted first before performing spirometry that required deep breaths to avoid the influence of forced expiration on IOS parameters (31).

Measurement of lung function was done by using spirometry. Spirometry was performed by a trained technician using *SpiroUSB (CareFusion Germany 23X)* in accordance with the American Thoracic Society (ATS) guideline. The technician would ensure that the subjects were in the correct position, the nose clip was in place, and that the subjects’ lips were sealed around the mouthpiece appropriately. Subjects must blow a minimum of 3 times, with a maximum of 8 repetitions depending on the quality of the test. They had to blow for a minimum duration of 6 s each. The acceptability criteria were when the difference between the two best readings was less than 5% and 150mls (29). Validated minimal clinically importance difference (MCID) for FEV_1_ in COPD has been established as per previous paper published, which is more than 100 mL improvement (34). MCID is used as a guide for physician to determine whether small changes in the outcome that is perceived as clinically benefited for patients after intervention (34, 35).

In order to reduce the risk of COVID-19 transmission and infection, subjects were required to perform a COVID-19 Rapid Test Kit-Antigen (RTK-Ag) or Reverse Transcriptase Polymerase Chain Reaction (RT-PCR) swab test 48 h prior to IOS and spirometry. Other measures taken to reduce COVID-19 transmission during the procedure included the use of level 3 personal protective equipment (PPE) and single-use, disposable mouthpieces.

CAT score questionnaires were used to determine the impact of COPD on the health of individual subjects which also reflected their quality of life (36). There was a correlation between CAT score with airflow limitation and GOLD classification as well (37). The CAT score increased as airflow limitation severity worsened. During recruitment, subjects were classified into four subgroups of impact level based on their CAT score: low impact (0–9), medium impact (10–20), high impact (21–30), and very high impact (31–40) (37). It was available in multi-language (Malay, English, and Chinese) depending on the subject’s preferences. We received permission from Mapi Research Trust (Special terms 78,299) for a user license. The MCID for CAT score value is by reduction of 2 points (35).

The 6MWT was a simple, practical test that required neither advanced training nor exercise equipment. The exercise capacity was measured by the distance a subject could walk in 6 min on a flat, hard surface. Prior to beginning a 6MWT, all subjects would need to rest in a chair for at least 10 min, and checked for their pulse, blood pressure, and pulse oximetry while they were resting (30). After they performed 6MWT, we would assess and record their walking distance and reevaluate any dyspnea or other new symptoms. In addition to measuring a subject’s functional status, the test had been used to predict morbidity and mortality (30). We did not perform 2 tests to account for learning effect due to limitation in manpower and time. The established MCID value for 6MWT in COPD subject is increment by 26 ± 2 meters. However, this data is only for severe COPD (34).

For this study, Aerobika^®^ OPEP would be administered to all subjects who met all inclusion criteria and would be instructed on how to use the device. The Aerobika^®^ OPEP device has been reported to improve sputum expectoration after 21 to 28 days of daily Aerobika^®^ utilization (23), exercise capacity by 6MWT (23, 24), spirometry result especially FEV_1_ (24), and FVC (23, 24), symptoms based on CAT score (24), and quality of life outcomes based on St. George’s respiratory questionnaire (SGRQ) (23, 24) in COPD patients. COPD patients who have experienced an exacerbation in the past are at a greater risk for future exacerbations (38), therefore prevention of exacerbation is an important component in managing COPD. From previous real-world studies, Aerobika^®^ has proven helpful to reduce rate of moderate-severe exacerbation (26, 39) and severe exacerbation in 30 days since post-discharge (25), with sustained lower rate of severe exacerbation in 12 months period (25). In the long run, other than providing clinical benefits, Aerobika^®^ is proven cost-effective in COPD management due to lesser exacerbation and hospital admission (39, 40). To date, there is no previous study has been done on the use of Aerobika^®^ OPEP to improve small airway resistance. There is also no study has been done to look for MCID for IOS improvement in subjects with COPD.

In our research, the subjects were instructed to use Aerobika^®^ for a total period of 24 weeks. They were required to inhale deeply and hold their breath for 2 to 3 s before exhaling (41). The subjects would then have to exhale actively, 3 to 4 times longer than inhalation through the device. They had to maintain a good seal on the mouthpiece and throughout breathing (41). After 10 to 20 breaths, subjects should perform 2 to 3 “huff” coughs to clear their airways (41). Duration and frequencies of Aerobika^®^ OPEP usage per day varies according to different studies (23, 24). In our study, subjects were advised to use Aerobika^®^ for 10 min for twice daily (41) preferably after inhalers, to enhance compliance. All subjects were also given an adherence diary to record each time they utilised Aerobika^®^ to increase compliance.

During week 12 and week 24 follow-ups, IOS, spirometry, and 6MWT were repeated. CAT scores and severe exacerbation events that required admissions were also recorded. In between follow up, regular video and phone calls were made every 2-weekly to ensure compliance with using Aerobika^®^ and to aid and consult should any problems arise.

### Statistical analysis

All statistical analyses were performed using the application Statistical Package for Social Sciences (SPSS) version 26.0. All variables’ distributional normality were evaluated using the Shapiro–Wilk test. For continuous variables with non-parametric distribution, the data were presented as medians and range (first quartile–third quartile) or variables with parametric distribution as means and standard deviation. The number of subjects (in %) was used for categorical variables.

The paired *t*-test was used to compare the means of continuous variables with normal distributions between two groups: pre- and post-interventional. Whereas Wilcoxon Signed Ranks Test was used to analyze continuous non-normally distributed variables. The McNemar’s test was used to compare nominal variables between the pre- and post-interventional groups. Statistical significance was declared when *p* < 0.05.

## Results

**Figure 1:**
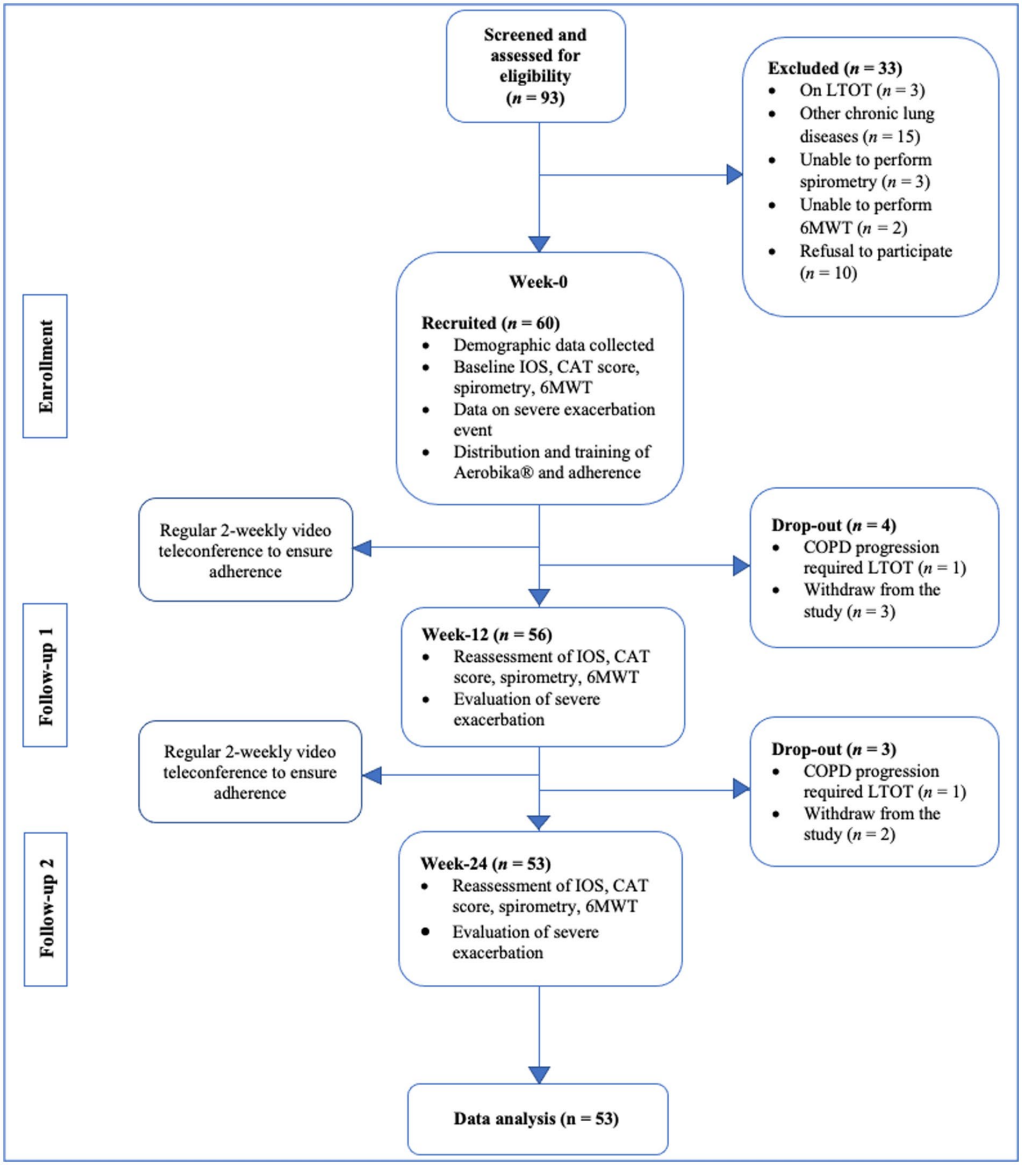
Study design and consort flow diagram.

Ninety-three subjects diagnosed with COPD were recruited from the respiratory clinic registry and screened for inclusion, and 60 subjects met the inclusion criteria. Thirty-three subjects were further excluded due to the following concerns: three subjects were using long-term oxygen therapy during night-time at home, 15 subjects had been diagnosed with chronic lung disease other than COPD, 3 subjects were unable to perform spirometry, 2 subjects unable to perform 6MWT, and another 10 subjects refused to participate in the study. Finally, 60 subjects completed all the baseline IOS, spirometry and 6MWT (Flow chart in Figure 1).

The mean age of the included subjects was 71.55 ± 6.33 years, with the majority of the subjects being male (96.7%). Among all participants, the majority was Malay (55.0%). They were predominantly ex-smokers (78.33%), 18.3% were still actively smoking, and only about 3.3% of the participants were a non-smoker. Among the active and ex-smokers, the median (IQR) smoking history was 45.00 (33.75–70.75) pack-year. The mean BMI was 24.59 ± 5.01 kg/m^2^. Of all the subjects recruited, 31.7% had no other medical comorbidities, whereas about 30.0% had 2 or more comorbidities. More than half of the subjects (53.3%) had medium CAT scores during recruitment, and 38.3% had high and very high CAT scores. The mean CAT score among all participants was 19.43 ± 7.10. The subjects predominantly had functional class grades 2 and 3 (38.3%) each, based on modified Medical Research Council (mMRC) grading. Other demographic characteristics of subjects, for example educational level, COVID vaccination status and inhalers type were further described in Table 1.

**Table 1:** Demographic characteristics of the study subjects.

Variables		All subjects, *n* = 60 *n* (%)
Age, years		71.55 (SD 6.33)^a^
BMI, kg/m^2^		24.59 (SD 5.01)^a^
Gender	Male	58 (96.7)
	Female	2 (3.3)
Ethnicity	Malay	33 (55.0)
	Chinese	24 (40.0)
	Indian	3 (5.0)
Education	Primary	20 (33.3)
	Secondary	29 (48.3)
	Tertiary	11 (18.3)
Smoking status	Non-smoker	2 (3.3)
	Active smoker	11 (18.3)
	Ex-smoker	47 (78.3)
Smoking history, pack-years (*n* = 58)		45.00 (33.75–70.75)^b^
COVID vaccination	1st vaccine	1 (1.7)
	2nd vaccine	6 (10.0)
	1st booster	53 (88.3)
	2nd booster	0 (0.0)
Comorbidities	No comorbidity	19 (31.7)
	DM	1 (1.7)
	HPT	19 (31.7)
	Heart disease	3 (5.0)
	DM + HPT	9 (15.0)
	HPT + heart disease	3 (5.0)
	DM + HPT + heart disease	6 (10.0)
COPD GOLD classification	A	4 (6.7)
	B	36 (60.0)
	E	20 (33.3)
COPD stages	II	34 (56.7)
	III	18 (30.0)
	IV	8 (13.3)
Duration of COPD	<10 years	48 (80.0)
	10–20 years	11 (18.3)
	>20 years	1 (1.7)
CAT score impact	Low (1–9)	5 (8.3)
	Medium (10–20)	32 (53.3)
	High (21–30)	18 (30.0)
	Very high (31–40)	5 (8.3)
mMRC	1	11 (18.3)
	2	23 (38.3)
	3	23 (38.3)
	4	3 (5.0)
Inhaled medications	Tiotropium	9 (15.0)
	FDC tiotropium/olodaterol	18 (30.0)
	FDC glycopyronium/indacaterol	4 (6.7)
	FDC formoterol/beclomethasone, + tiotropium	5 (8.3)
	FDC salmeterol/fluticasone, + tiotropium	11(18.3)
	FDC tiotropium/olodaterol, + budesonide	10 (16.7)
	FDC glycopyronium/indacaterol, + budesonide	2 (3.3)
	FDC formoterol/budesonide, + tiotropium	1 (1.7)

DM, diabetes mellitus; HPT, hypertension; heart disease, includes ischemic/ non-ischemic cardiomyopathy, heart failure, valvular heart disease; mMRC, modified Medical Research Council; FDC, fixed-dose combination.

a Mean (SD).

b Median (IQR).

### Parameters of IOS, spirometry, 6MWT and CAT score at 0-, 12-, and 24-week

In Table 2, it shows the baseline data of IOS, spirometry, 6MWT and CAT score at week-0. All subjects were confirmed COPD based on spirometry with a median (IQR) FEV_1_/FVC ratio of 52.31 (44.67–61.67) %. The mean FEV_1_ was 1.22 ± 0.41 L with 52.15 ± 15.65% predicted. This made up more than half of the subjects in stage II COPD severity according to GOLD guideline (1). The median (IQR) value of R5–R20 which reflected small airway resistance was 2.69 (2.06–4.19) cmH_2_O/L/s. Majority of the study subjects were symptomatic with mean CAT score of 19.43 ± 7.10. The mean 6MWT among all subjects were 213.23 ± 88.78 meter during baseline.

**Table 2:** The parameters of IOS, spirometry, 6MWT and CAT score at 0-, 12-, and 24-week.

	0-week (Baseline)	12-week	24-week	*p*-value (0–12 week)	*p*-value (12–24 week)	*p*-value (0–24 week)
R5, cmH20/L/s	5.97 (4.90–7.67)^b^	5.82 (4.69–7.67)^b^	5.60 (4.27–7.42)^b^	**0.008** ^c^	**0.007** ^c^	**<0.001** ^c^
R5, %_pred_	192.49 (170.65–253.16)^b^	191.32 (158.64–245.34)^b^	173.26 (143.55–240.55)^b^	**0.007** ^c^	**0.007** ^c^	**<0.001** ^c^
R20, cmH20/L/s	3.25 (2.89–3.83)^b^	3.18 (2.81–3.97)^b^	3.07 (2.71–3.71)^b^	0.488^c^	**0.040** ^c^	**0.049** ^c^
R5–R20, cmH20/L/s	2.69 (2.06–4.19)^b^	2.54 (1.74–3.88)^b^	2.45 (1.64–3.40)^b^	**0.021** ^c^	**0.014** ^c^	**<0.001** ^c^
AX, cmH20/L	28.88 (21.28–41.55)^b^	26.67 (20.62–38.83)^b^	25.07 (15.90–38.11)^b^	0.293^c^	0.066^c^	0.072^c^
X5, cmH20/L/s	−3.47 (SD 1.40)^a^ −3.41 (−4.39 to −2.44)^b^	−3.52 (−4.37 to −2.50)^b^	−3.26 (SD 1.34)^a^ −3.31 (−4.25 to −2.23)^b^	0.854^c^	0.085^c^	0.214^d^
FEV_1_/FVC, %	52.31 (44.67–61.67)^b^	53.00 (47.00–64.00)^b^	55.00 (47.50–65.00)^b^	**0.008** ^c^	0.950^c^	**<0.001** ^c^
FEV_1_, L	1.22 (SD 0.41)^a^	1.29 (SD 0.45)^a^	1.35 (SD 0.48)^a^	**0.018** ^d^	0.182^d^	**0.001** ^d^
FEV_1_, %_pred_	52.15 (SD 15.65)^a^	54.78 (SD 16.39)^a^	57.27 (SD 17.31)^a^	**0.025** ^d^	0.118^d^	**0.001** ^d^
FVC, L	2.35 (SD 0.74)^a^ 2.23 (1.78–2.79)^b^	2.36 (SD 0.72)^a^ 2.34 (1.84–2.84)^b^	2.31 (1.98–2.96)^b^	0.938^d^	0.183^c^	0.475^c^
FVC, %_pred_	67.87 (SD 18.81)^a^ 63.5 (54.25–80.00)^b^	68.13 (SD 17.75)^a^ 66.00 (53.00–79.50)^b^	68.0 (57.50–79.50)^b^	0.975^d^	0.137^c^	0.311^c^
FEF_25–75_, L	0.46 (0.35–0.82)^b^	0.55 (0.38–0.76)^b^	0.57 (0.39–0.94)^b^	**0.023** ^c^	0.545^c^	**0.002** ^c^
FEF_25–75_, %_pred_	22.00 (15.00–34.50)^b^	25.00 (16.25–32.00)^b^	27.00 (17.50–39.00)^b^	**0.024** ^c^	0.260^c^	**<0.001** ^c^
6MWT, meter	213.23 (SD 88.78)^a^	221.89 (SD 92.86)^a^	243.09 (SD 103.33)^a^	0.267^d^	**0.043** ^d^	**0.016** ^d^
CAT score	19.43 (SD 7.10)^a^ 19.00 (15.00–24.00)^b^	13.46 (SD 5.98)^a^ 14.00 (9.25–16.75)^b^	9.00 (7.00–14.00)^b^	**<0.001** ^d^	**< 0.001** ^c^	**< 0.001** ^c^

a Mean (SD).

b Median (IQR).

c Wilcoxon signed ranks test.

d Paired *t*-test.Bold indicates statistically significant.

Following intervention with Aerobika^®^ OPEP, there was a significant improvement in the primary variable outcome of small airway parameters, particularly R5–R20 as described in Table 2. There was also reduction in R5 and R20 value at the end of 24 weeks of study period. Other secondary outcomes results also showed improvement after Aerobika^®^ OPEP intervention, e.g., lung functions (FEV_1_, FEF_25–75_), 6MWT and CAT score (Table 2).

We also look at the median difference (IQR) of changes following Aerobika^®^ use after 12 weeks comparing 24 weeks. There was significant improvement in IOS parameters particularly R5–R20, R5, as well as R20 as shown in Table 3.

**Table 3:** The median difference in IOS parameters between 0- to 12 weeks and 0- to 24 weeks of Aerobika^®^ OPEP usage.

	0- to 12-week	0- to 24-week	*p*-value
R5, cmH20/L/s	−0.28 (−0.90 to 0.22)	−0.83 (−1.46 to 0.06)	**0.007**
R5, %_pred_	−9.58 (−29.83 to 7.03)	−28.23 (−49.17 to 1.96)	**0.007**
R20, cmH20/L/s	−0.05 (−0.40 to 0.27)	−0.14 (−0.49 to 0.17)	**0.040**
R5–R20, cmH20/L/s	−0.27 (−0.71 to 0.12)	−0.76 (−1.30 to −0.05)	**0.014**
AX, cmH20/L	−1.66 (−6.78 to 4.46)	−4.94 (−9.71 to 5.45)	0.066
X5, cmH20/L/s	−0.07 (−0.52 to 0.48)	0.18 (−0.82 to 1.22)	0.085

Data are displayed as median (IQR). Data are analysed using Wilcoxon signed ranks test.Bold indicates statistically significant.

### Severe exacerbation events before and after using Aerobika^®^ OPEP

We also analysed severe exacerbation events 24 weeks before and after Aerobika^®^ OPEP. Out of 60 subjects recruited, 19 (31.7%) of them had severe exacerbation events that required hospitalisation 24 week before Aerobika^®^ OPEP intervention. Our study found 15 severe exacerbation event discordant pairs 24 weeks before and after Aerobika^®^ use. Out of 17 subjects with severe exacerbation (hospital admission) pre-intervention, there were 11 subjects that did not have hospital admission for exacerbation during the study period. Whereas, out of 36 subjects without any hospitalisation before Aerobika^®^ administration, 4 subjects developed severe exacerbation during 24 weeks follow up. However, it was not statistically significant (Table 4).

**Table 4:** Severe exacerbation events 24 weeks before and after Aerobika^®^ OPEP intervention.

Severe exacerbation events, *n*		24 weeks after Aerobika^®^		Total	*p*-value
		No	Yes		
24 weeks before Aerobika^®^	No	32	4	36	0.118
	Yes	11	6	17	
Total		43	10	53	

Data are analysed using McNemar’s test. The total number of subjects analysed was 53 (before and after Aerobika^®^ administration).

## Discussion

Our study demonstrated that the use of Aerobika^®^ OPEP improved small airway disease in COPD subjects. This was primarily through a significant decrease in R5–R20 after 12 weeks of Aerobika^®^ use, with sustained reduction after 24 weeks. When we compared the median difference between 12-week and 24-week interval usage from baseline, the difference was also statistically significant, indicating that prolonged use of Aerobika^®^ OPEP would have a more significant positive effect on small airway parameters. There is no established MCID yet for IOS parameters in COPD subjects. In previous paper, the proposed MCID for IOS parameters were mainly for asthmatic subjects, in which decline of ≥0.06 kPa/L/s (≥0.61 cmH_2_O/L/s) for frequency dependence of resistance (FDR) and ≥ 0.65 kPa/L (≥6.63 cmH_2_O/L) for AX, respectively, considered to be significant (42).

Other parameters in the IOS, R5 absolute and percentage predicted value were also reduced significantly and sustainable over 12 and 24 weeks of Aerobika^®^ use. The improvement on R20 could only be seen significantly after 24 weeks of Aerobika^®^. However, no significant changes in the value of AX and X5 parameters were demonstrated in this study.

We demonstrated improvement in our subjects’ FEV_1_ absolute value and FEV_1_ percentage predicted value after 24 weeks Aerobika^®^. This corresponded well with prior research done by Gupta et al. (24) which proved Aerobika^®^ would help to improve lung function particularly FEV_1_. In our subjects, there was improvement of 130 mL in FEV_1_ value at the end of 24 weeks study period, in which achieved the MCID for FEV_1_ improvement in COPD subjects.

FEF_25–75_ measurement, could be the earliest abnormality seen from spirometry to indicate small airflow limitation even in subjects with normal spirometry (43, 44) and aid in the early detection and diagnosis of COPD, allowing for the initiation of treatment as soon as possible. FEF_25–75_ measurement in our study showed significant improvement after treatment with Aerobika^®^. This would coincide with the previously mentioned significant enhancement of small airway resistance parameter. Other than that, previous research done by Svenningsen et al. and Gupta et al. (23, 24) had shown that Aerobika^®^ could improve FVC. Our study also showed improvement in FVC parameters throughout the 24 weeks study, even though it was not statistically significant.

Our study also demonstrated an improvement in exercise capacity, as measured by a significant increase in 6MWT after 24 weeks of Aerobika^®^ administration. In our subjects, there was significant improvement in 6MWT by 29.86 meters, and achieved the MCID for severe COPD (26 ± 2 meters) (34). There is no validated MCID for 6MWT in moderate COPD subjects. The improvement of 6MWT in our subjects was also supported by other studies conducted previously (23, 24).

In the present study, the CAT score was used to evaluate symptoms. Few previous studies had demonstrated significant improvement in COPD symptoms, as indicated by a decline in CAT score during follow-up (24). We were also able to demonstrate a significant reduction in COPD symptoms in our study by showing a sustained reduction in the CAT score values over 12 and 24 weeks. Our study showed reduction of CAT score by 10 points at the end of 24 weeks study period, and achieved the MCID for CAT score reduction by 2 scores (35).

In previous studies done by Tse et al. (25), following Aerobika^®^ usage, there was reduction of severe exacerbation up to 1 year after discharge from hospital. In our study, despite the reduction in severe exacerbation and hospitalisation in our subjects when comparing 24 weeks before and after receiving Aerobika^®^, this reduction was not statistically significant. This might result from only stable COPD subjects recruited for our study. A shorter follow-up period might also account for the non-significant severe exacerbation outcome. In our study we failed to reject null hypothesis regarding exacerbation event, e.g., there is no difference of exacerbation events 24 weeks before and after Aerobika^®^ OPEP intervention. However the possibility of type 2 error is minimized as we achieved the sample size required. There is also no validated MCID established for severe exacerbation event in COPD subjects (34).

To our knowledge, this was the first study to evaluate the effectiveness of Aerobika^®^ OPEP in improving small airway disease in COPD subjects. Our postulation regarding all the positive results was that Aerobika^®^ OPEP improved airway resistance by facilitating airway clearance. The enhancement of small airway resistance was subsequently accompanied by enhancements in symptoms, spirometry, and exercise capacity.

There were a few identified limitations in this study. This was a single-centre study; hence the total number of participants was limited. There was also the possibility of compliance and adherence issues with Aerobika^®^. Even though we conducted regular video teleconferences to improve adherence, there was still a possibility that subjects would not adhere to Aerobika^®^ on days when they were not supervised. A further limitation identified in this study was that each subject might have a different understanding and perception of the CAT score, which may introduce response bias. Another limitation of our study was that, this was a single-arm and not a randomized controlled trial. There might also be confounding factors that could interfere with the results of study; for example usage of mucolytic agents during study period. However, all of the subjects that were using mucolytics were only prescribed with bromhexine by the attending doctor, and none were using mucolytic agents with antioxidant properties, e.g., N-acetylcysteine and erdocysteine as per COPD GOLD guideline (1). Other than that, our study required all the subjects involved to use Aerobika^®^ OPEP on a fix amount of time (10 min, twice per day) rather than a specific number of repeats. Thus, this number of repeats might not be adequate for some subjects who were more symptomatic.

## Conclusion

In conclusion, Aerobika^®^ OPEP was advantageous for the management of COPD. It demonstrated significant improvement of small airway parameters as early as 12 weeks of use, with sustained improvement over 24 weeks. Aerobika^®^ OPEP administration also had significantly improved lung function (FEV1, FEF_25–75_), 6MWT, and CAT scores over 24 weeks. We suggest future evaluation of small airway parameters involving multicentre and larger sample sizes, which may help to validate the results and solidify its role in the management of COPD, other than helping to identify predictors on small airway disease subjects that will benefit most from Aerobika^®^ OPEP intervention. A future research evaluating effectiveness of Aerobika^®^ OPEP using different frequencies and duration of use per day may guide physicians on the optimum usage of this device. We recommend that this device be incorporated in the COPD guidelines as an add on therapy in patients with small airway disease.

## Data availability statement

The raw data supporting the conclusions of this article will be made available by the authors, without undue reservation.

## Ethics statement

The studies involving human participants were reviewed and approved by Research Ethics Committee, Universiti Kebangsaan Malaysia (FF-2021-175). The patients/participants provided their written informed consent to participate in this study.

## Author contributions

MFAH: conceptualization, supervision, writing, reviewing, and editing manuscripts. SNS, AAA, AB, MFMJ, and MFAH: data curation. SNS and SAS: formal analysis. SNS, MFMJ, AAA, and MFAH: funding acquisition and methodology. SNS, MFAH, MFMJ, NN, NBH: project administration. SNS: writing-original draft. All authors contributed to the article and approved the submitted version.

## Funding

The authors received fundamental grant from Universiti Kebangsaan Malaysia with research code FF-2021-175.

## Conflict of interest

The authors declare that the research was conducted in the absence of any commercial or financial relationships that could be construed as a potential conflict of interest.

## Publisher’s note

All claims expressed in this article are solely those of the authors and do not necessarily represent those of their affiliated organizations, or those of the publisher, the editors and the reviewers. Any product that may be evaluated in this article, or claim that may be made by its manufacturer, is not guaranteed or endorsed by the publisher.
